# Retraction Note: Visualising the dynamics of live pancreatic microtumours self-organised through cell-in-cell invasion

**DOI:** 10.1038/s41598-024-76022-8

**Published:** 2024-10-22

**Authors:** Yukiko Miyatake, Kaori Kuribayashi-Shigetomi, Yusuke Ohta, Shunji Ikeshita, Agus Subagyo, Kazuhisa Sueoka, Akira Kakugo, Maho Amano, Toshiyuki Takahashi, Takaharu Okajima, Masanori Kasahara

**Affiliations:** 1 Department of Pathology, Faculty of Medicine and Graduate School of Medicine, Hokkaido University, Sapporo, Japan; 2 Institute for the Advancement of Higher Education, Hokkaido University, Sapporo, Japan; 3 Graduate School of Information Science and Technology, Hokkaido University, Sapporo, Japan; 4 Creative Research Institution Sousei, Hokkaido University, Sapporo, Japan; 5 Faculty of Science, Hokkaido University, Sapporo, Japan; 6 Research Development Section, Hokkaido University, Sapporo, Japan; 7 Department of Pathology, Hokkaido Gastroenterology Hospital, Sapporo, Japan

Retraction of: *Scientific Reports* 10.1038/s41598-018-32122-w, published online 19 September 2018

Editors have retracted this Article.

After publication of this Article, the Authors contacted the Editors to note that, contrary to the statement in the paper, the use of patient samples for IHC experiments was not approved by the Medical Ethics Committee of Hokkaido University Faculty of Medicine, and requested that the Article be retracted.

All Authors agree to this retraction.

